# Tri-colour concept with the use of LMA CTrach

**DOI:** 10.4103/0019-5049.79880

**Published:** 2011

**Authors:** Geetanjali Chilkoti, Medha Mohta

**Affiliations:** Department of Anaesthesiology and Critical Care, University College of Medical Sciences and Guru Teg Bahadur Hospital, Delhi, India

Sir,

In the past few years, a new optical intubation device, i.e. laryngeal mask airway (LMA) CTrach has emerged as a useful alternative for the facilitation of tracheal intubation in difficult airway situations provided the interdental distance is ≥25 mm.[1] The LMA CTrach is functionally identical to intubating laryngeal mask airway (ILMA), but in addition has an integrated fibreoptic bundle with liquid crystal display (LCD). This system enables ventilation and allows real-time visualization of endotracheal intubation.[2]

The LMA CTrach is inserted in neutral head position using one handed rotational technique.[2] Following confirmation of the adequate lung ventilation, the viewer is connected to the CTrach and the laryngeal structures are visualized. After obtaining the best laryngeal view, the tracheal tube is passed through the barrel of the CTrach and tracheal intubation is facilitated under direct vision. However, the most common problem encountered with the use of LMA CTrach is the frequent requirement of application of corrective manoeuvres to obtain the best laryngeal view which ultimately determines the success of intubation.[3] The causes of poor view of vocal cords include down folding of the epiglottis, contact with the lens, secretions or fogging of the lens during insertion.[1] If complete view of laryngeal structures is not achieved, the corrective manoeuvres are applied according to the likely causes of poor view which are listed in Table 1. These manoeuvres include the technical adjustments, e.g. changes in focus and light intensity and the positional adjustments, e.g. changes in the depth of insertion, external manipulation of the larynx, down-up-down manoeuvre and Chandy’s manoeuvre.[1] For down-up-down manoeuvre, the inflated CTrach is slightly pushed down, in case of no improvement in view it is slowly withdrawn from the pharynx by about 6 cm while watching the monitor and then reinserted. This manoeuvre helps in adjusting the depth of insertion and also unfurling the epiglottis.

**Table 1:** Tri-colour concept

Colour	Causes	Prevention	Management
Red (with partial or no laryngeal view)	Lens touching the mucosa or epiglottis Presence of blood	Correct size selection Gentle approach	Down-up-down manoeuvre Suction out the blood /secretions and clean the lens if required
White	White secretions	Antisialogogue therapy before CTrach insertion	Suction out the secretions Remove, clean and reinsert the CTrach
Black	Low light intensity	Adjust the light intensity before start of procedure	Adjust the light intensity
	Inadequate depth of insertion		Adjust the depth of insertion

On initial insertion, if the complete laryngeal view is not seen on the viewer, the presence of three distinct colours, i.e. red, white and black can indicate the likely causes of inadequate view. The knowledge of this tri-colour concept described below can be extremely helpful in obtaining the best laryngeal view and performing intubation successfully through the LMA CTrach

Once the best laryngeal view is achieved, the application of medial-lateral manoeuvre can help in centralizing the glottic aperture on the viewer and finally the application of Chandy’s manoeuvre (using the CTrach handle to lift the CTrach cuff away from the posterior pharyngeal wall) at the time of intubation can eventually influence the success of first attempt intubation.[4]

The achievement of the best laryngeal view and facilitation of tracheal intubation through the LMA CTrach can be made simpler by understanding of this tri-colour concept. We feel that the learning of tri-colour concept should be an integral part of the teaching of CTrach use as it can help in increasing the efficacy of this useful airway device in difficult airway situations.
